# The relationship between musicianship and pain. Is chronic pain and its management a problem for student musicians only?

**DOI:** 10.3389/fpain.2023.1194934

**Published:** 2023-09-06

**Authors:** Michaela Korte, Deniz Cerci, Roman Wehry, Renee Timmers, Victoria J. Williamson

**Affiliations:** ^1^ Department of Music, The University of Sheffield, Sheffield, United Kingdom; ^2^ Universitätsmedizin Rostock, Klinik für Forensische Psychiatrie, Zentrum für Nervenheilkunde, Rostock, Germany; ^3^ Helios Klinikum Hildesheim, Akademisches Lehrkrankenhaus der Medizinischen Hochschule Hannover, Hildesheim, Germany; ^4^ Independent Academic, Sheffield, United Kingdom

**Keywords:** pain catastrophizing, chronic pain, music students, emotions, pain-related behavior

## Abstract

**Introduction:**

The neuro-biological side of chronic pain research has presented reliable evidence of distinct cortical and spinal alteration compared to healthy individuals. Furthermore, research suggests that musicians are especially vulnerable to pain, and recent neurological investigations into musicians' brain plasticity support this hypothesis. However, chronic pain is not acute pain plus time, but a separate condition, and little is known about musicians' chronic pain-related emotions and behaviors. This knowledge, however, is a crucial step in understanding how chronic pain is processed by musicians.

**Methods:**

This study investigated pain catastrophizing as a critical pain-related behavior and emotional concept alongside six complementary variables: anxiety, depression, depersonalisation, burnout, coping strategies and professional identity.

**Results:**

103 under- and postgraduate students from various higher education institutions participated in an online survey. Students were allocated into three groups according to their main study subject and type of institution: music college musicians, university musicians and university non-musicians. A tree model confirmed the current chronic pain multifactorial model, suggesting a combination of several variables before catastrophizing pain. Group testing, however, showed that university non-musicians' pain catastrophizing was significantly worse especially when compared to music college musicians. Music college musicians and university musicians were less prone to maladaptive pain processes, despite perceiving pain for significantly longer.

**Discussion:**

This novel finding indicates that chronic pain does not inevitably lead to dysfunctional pain processing for musicians and should be reflected accordingly to optimize pain-control. The biopsychosocio model of chronic pain provides a robust framework for future research in this population.

## 1. Introduction

Music making appears superficially to be intrinsically linked to pain and its neuropsychological impacts, at least for (aspiring) professional musicians. Physical risk factors for the playing of various instruments, as well as for singers, have been well documented, and preventative pain advice was offered as early as 1700 (1, 2). Composers have invested (their) physical pain into their musical compositions as part of opera or song and/or have crafted their works to accommodate their personal injuries whilst still performing at the highest level (3, 4). Thus, people have argued successfully that outstanding artists and composers who do not suffer from pain are rather the exception, and that happy individuals create nothing (5, 6). This argument seems to have taken roots within the musician community and has led to the superstition that painful conditions and/or suffering are a consequence of life as a professional musician. Is this line of argument an accurate reflection of today's musicians?

At first glance the answer might appear to be yes. Research concurs that reports of pain, mostly focused on musculoskeletal pain perception, are significantly higher in groups of musicians compared to the general population (7, 8). Based on such a simple comparison one might predict that musicians would be disproportionally affected by pain (7, 8) However, if we want to further deepen the understanding of pain in musicians, we have to turn to studying the lengths of time that pain was perceived by musicians. A duration of longer than three months is the first evidence that pain is no longer acute in nature but can be considered chronic pain.

General research into pain has long established these two distinct concepts: acute pain and chronic pain. Acute pain, an unpleasant sudden onset sensory and emotional experience, acts as a vital alert or warning mechanism, for instance to distinguish (the extent of) an injury or to detect infections. Acute pain can indicate physical damage to the organism. However, the assessment of any pain, is not exclusively based on the perception of said pain. Both concepts of pain have no proportional relationship between the injury and the perceived pain, although this sentence carries more relevance for chronic pain than for acute pain (9). During normal healing time, endogenous mechanisms such as endorphins, GABA, or monoaminergic pathways and systems all operate as preventative measures and prevent acute pain from transforming into chronic pain (10). One reason for developing chronic pain is the failure of these mechanisms.

Pain that persists or recurs longer than three months is considered chronic (11, 12), and can become the predominant problem following injury or infection. “Chronification”, the development of acute pain into chronic pain, is essentially the process of transient pain becoming persistent (13). Biological, psychological, and social factors all contribute to the multifactorial model of chronic pain. The condition is characterized by significant emotional distress (e.g., anxiety, depressive mood) and/or functional disabilities (e.g., reduced social participation, interference in daily life). Thus, chronic pain is not the prolongation of acute pain, but a separate, complex state of body and mind that has distinct characteristics, and is consequently understood and coded in the International Statistical Classification of Diseases and Related Health Problems (ICD) as a separate condition to acute pain (e.g., R52.1 or R52.2 or F45.41; the number of codes for chronic pain depend on origin and composition of symptoms). When it is chronic, pain-related injury is only marginally defined by diagnosable physical damage and this type of pain no longer carries a warning function (12, 14). This pain does, however, warrant specific diagnostic evaluation, therapy, and rehabilitation.

Mechanisms of pain chronification include (neuro)biological changes (e.g., thalamus, limbic system and cortical structures, depending on the location of the pain), emotions (e.g., depression, anxiety or burnout), and pain-related beliefs and cognitions (i.e., pain-related inner monologues or meta-cognitions developing across time that lead to a modified behavior, maladaptive strategies such as learned helplessness/hopelessness or pain catastrophizing). Pain catastrophizing is a passive maladaptive coping strategy, a combination of rumination, magnification of pain, and the feeling of being helplessly exposed to pain. The pain-related feelings/beliefs are independent of the actual (physical) pathology and are shaped mostly by the individual. The preoccupation with pain can become so intense that it becomes a focus for life, propelling individuals deeper into despair. For individuals with high levels of pain catastrophizing it is extremely difficult to reach out for therapy/treatment. Pain catastrophizing has thus emerged as one predictor for early retirement (15).

Based on the existing literature on musicians and pain, we can assume that the pain described is mostly chronic. Firstly, the duration of (musculoskeletal) pain is generally noted as 12 months and longer (8). Most often, this pain is due to incorrect postures. In studies with music students postures were worse compared to non-music students (i.e. without instruments) and degraded significantly during music making (16). This is especially prominent for asymmetric instruments such as violin or flute. Secondly, we have neurobiological indications of pain-based changes in musicians, such as (hyper-) sensitivity of the nociceptive system and differences in cortical neuroplasticity, which are in line with pain chronification processes (17, 18).

However, there is a notable discrepancy between the musculoskeletal pain reported by professional orchestra musicians and the actual identifiable physical dysfunctions diagnosed by physicians in these musicians (19). While this shows evidence that a great deal of the pain perceived by musicians is chronic, based on neurobiological indicators, we have little to no evidence of how this pain is processed. This discrepancy might sound minor compared to the wealth of complex neurological evidence: pain processing behaviours and emotions are, however, a crucial knowledge step towards understanding the pain chronification process in musicians.

Summarizing these findings, pain prevalence is high in professional musicians compared to their peers. Based on the pain durations reported, this process starts at least as early as training (music) college. There is sufficient evidence that a great deal of the pain musicians perceive can be considered chronic. While there are few direct investigations into pain-related emotions and behaviours, there is conflicting evidence on variables/predictors that enhance or prevent chronification (7, 20). Moreover, due to a lack of studies into musicians' chronic pain as compared to other professions, there is the question of proportions: are musicians disproportionally affected by chronic pain compared to their peers and, if so, how early in their training can we assess a difference? This can be conceptualized as a research query: is being a student musician a significant risk factor contributing to catastrophizing pain?

The present study investigated pain catastrophizing as one of the most important pain-related factors in young training musicians. The results contribute to answering the following questions: (1) how does pain catastrophizing compare between student musicians and student non-musicians? (2) Do the chosen degree/course and subsequent career prospects matter when it comes to how/if pain is catastrophized? (3) What role (if any) do more general pain chronification predictors, such as depression, burnout, or professional identification, play in the chronification process?

In endeavoring to answer these questions, this study will determine if and/or how pain catastrophizing influences young student musicians and non-musicians and how or if they differ between groups, their professional identification as musicians and subsequent career prospects. To inform our choice of factors, we concentrated on previously identified and well-evidenced pain chronification predictors, such as anxiety, depression, professional identity, depersonalization, burnout, coping styles and sleep (13, 21, 22). The outcomes of this study provide timely evidence on how we may better identify (music) students, who are at risk of chronic pain during their training years.

## 2. Material and methods

This exploratory study used a predictive design and random sampling in order to find combinations of variables that would most likely determine a change in pain catastrophizing.

### 2.1. Procedure

Participants were recruited online via the students' servers. This means that an invitation for participation was emailed to every student in the specific institutions as part of their regular newsletter. This study was carried out in accordance with the recommendations of the university and the protocol was approved by the Ethics Committee of the Department. All participants had to be at least eighteen years of age and to study in higher education. They gave written informed consent.

#### 2.1.1. Statistical analysis

Evaluation of the data was performed using RStudio (2022.12.0) (23) and G*Power (24). Power of *β* = .8 was considered as appropriate. Power calculations found the minimum for pairwise comparison with an expectation of non-linear distribution to be 96 participants in total (33 participants per group). Missing values were imputed using the package Hmisc. Rpart and rpart.plot were used for the tree model to analyse predictability. *χ*^2^, Mann–Whitney *U*-test and ANOVA were used for (pairwise) comparisons. Bonferroni corrections were applied to safeguard against multiple testing, and Spearman's correlation for correlations. Tree methodology is used to classify data based on multiple covariates or develop predictors for a target variable. This approach is robust as the algorithm does not impose a parametric structure, and can deal with large, complicated data sets. Contrary to linear models, tree models do not presume a functional linear relationship between variables. The decision tree's algorithm finds the best splits of the data, so that the entropy is maximized. Tree model partition observations split the dependent variable (pain catastrophizing) into homogeneous sub-groups, where the observations have a similar value (i.e., low, moderate or high anxiety scores). Each node of the tree represents the independent variable that maximally increases the homogeneity of the data for splitting. This splitting process is repeated until it reaches either the group size criterion, or until further splitting would not increase homogeneity (for a detailed introduction to tree methods, see Breiman et al. (25); for an overview/use in music psychology and medicine, see Müllensiefen (26); Weng et al. (27).

### 2.2. Material

Participants were asked to share demographic details such as age, relationship status, pain perception, etc (see Table 1).

**Table 1:** Demographics.

	Music College Musicians	University Musicians	University Non-Musicians
Age (mean, SD)	27.9; 8.74*	21.6; 3.44	23.3; 7.85
Family/relationship status: stable	57%	49%	48%
Family/relationship status: single	43%	51%	52%
Undergraduate/Postgraduate^a^	8/12	18/16	19/13
Stress relief, active (running, yoga, etc.)^b^	63%	15%	87%
Stress relief, other (meditation, therapies, etc)^b^	9%	67%	7%
Regular practice time (years)^c^	4.76	4.25	0
Regular practice time (hours per day)	6.14	2.45	0
Music theory lessons (years)	4.82	4.82	0
Formal instrumental/vocal training (years)	6.11	4.74	0
Attending live concerts^d^	5.35	3.22	1.05
Attentively listening to music^e^	30–60 min	30–60 min	0–15 min

^a^ Missing data due to non-disclosure, observation based on combination of demographic data (e.g., highest school/university qualifications).

^b^ Regular activities; once or twice a week over a minimum period of two months.

^c^ GOLD-MSI gives ranges and not an exact number, e.g., 4–6 years.

^d^ Member of audience, during past year.

^e^ Average per day.

* Missing data, see limitations for explanation.

The Örebro Musculoskeletal Pain Screening (28) measures how participants' rate their level of catastrophizing pain, for instance based on how their experience of pain affects their performance at work/higher education. In 21 questions it addresses pain beliefs and expectations. The higher the score, the more likely it is that the individual will remain disabled by pain, unable to return to work. The authors specified that, using a six-months prediction, 71% of patients were correctly classified (sensitivity, 72%; specificity, 70%), with a high reliability (*α* = .97, *p* ≤ .05) and a high internal consistency (*α* = .87). A score of 105 indicates a moderate risk, ≥130 a high risk of being disabled by pain.

The Hospital Anxiety and Depression Scale (HADS) (29) collects information on depression and anxiety symptoms using two separate scales (7 items per scale) based on a 4-point Likert response (Cronbach's *α *= .6). The HADS discriminates well between anxiety and depression. It is a good fit to the Rasch Model, stable across professions and less vulnerable to cultural bias. The cut-off for significant depression and anxiety was set at ≥ 9.

The Cambridge Depersonalisation Scale (CD-9) (30) covers depersonalization through 9 questions. This scale has shown adequate internal consistency and temporal stability (*α* = .92, retest reliability 10–14 days: *r_tt_* = .86). Scores are added up and can reach 0–90, with 0 indicating no depersonalization. The cut-off point for significance was set at the level requested by the scale's authors at ≥19 (short, transient) and ≥90 (unique condition).

The Brief COPE (31) distilled the 14 scales from the original questionnaire into three scales (28 questions). This allows for diverse testing of stress coping and correlation of findings. The three scales are: active functional coping (e.g., “I actively did something”), functional cognitive coping (e.g., “I tried to find something positive in what happened.”) and dysfunctional coping strategies (e.g., “I used alcohol/other substances to help me through this situation”). Internal consistency was found to be good for all subscales: emotion-focused, problem-focused, and dysfunctional subscales (*α* = 0.72, 0.84, 0.75).

The Copenhagen Burnout Inventory, (CBI) (32) consists of three main scales: personal burnout, work burnout, and client-related burnout. The authors' attested all three scales to have very high internal reliability (*α* = .85–87). This study's design was modelled on the study by (33) to reflect the dual client burnout problem of students: the client questions were doubled up, exchanging the word client with fellow student in one set, and professor in the other. These scores reflect the level of exhaustion and fatigue perceived from this interpersonal relationship that derives from the students' interaction with fellow students and/or academic staff (professor).

The Athletic Identity Measurement Scale (34) is a 10-item scale that assesses the strength and exclusivity of professional identity. The higher the score, the more a candidate identifies with being an athlete (10–70, mean of 40; internal consistency of *r *= .93; test-retest reliability of *r *= .89. We used Vitale's (35) adaptation for musicians, changing the word athlete to musicians and called the questionnaire Musicians Identification Measurement Scale (MIMS).

The Goldsmiths Musical Sophistication Index (Gold-MSI) (36) is a self-reported test that assesses an individual's propensity to engage with music. It is modelled on multidimensional construct of musical sophistication. With Cronbach's *α* = .914 the scale is suitable instrument. We used two of the test's subscales: active engagement and musical training (7 and 9 questions).

The Pittsburgh Sleeping Quality Index (PSQI) (37) measures subjective quality of sleep (9 questions). The sleep questionnaire was administered to the music college musicians only. All participants in this group had regular evening performance schedules compared to the other groups which gave rise to the idea that lack of sleep could influence their pain perception and management. It has a high test-retest reliability and a good validity for different age groups (Cronbach's Alpha *α* = .69). The PSQI was able to correctly identify 88.5% of all patients and controls, representing a 89.6% sensitivity and a 86.5% specificity rate, using an empiric cut-off point of 5. We hypothesized music college students would have greater prevalence in maladaptive pain processing, which includes sleep disturbances.

### 2.3. Results

103 under- and postgraduate students [75% United Kingdom (UK), 16% European Union countries, 9% United States of America; age mean = 23.6 years] from various institutions and with different primary study subjects (62% music, 38% medicine, psychology, biology) participated in this study. 67 students were from the University of Sheffield and 36 students from various music colleges. The UK offers two ways of studying music: at music college/conservatoire, a route preferred by those aiming to attain elite music status, and university which offers a more academic approach to the subject. Similar to elite college athletes, age is crucial for music college students in classical music. Auditions and competitions on national and international levels have a strict age limit, thus making it imperative to start the musical education early. All participants were divided into three groups. The selection was made based on their place of study (music college or university) and their engagement with music (for university students only). The students from music colleges thus remained together in one group. The group of university students was divided into two sub-groups: firstly, the university musicians' group which comprised students who self-identified as musicians irrespective of their main study subject (music or science). These participants showed equal levels of years of practice, lessons taken and engagement with music as the group of students from music college and comprised 31 participants (see Table 1). Secondly, university students who hardly showed any engagement with music (no instrumental or theory lessons) constituted the non-musicians' group. This group had 36 participants. For ease of reference, the three different groups will from now on be referred to as university musicians, university non-musicians, and music college musicians (see all tables below).

#### 2.3.1. Scale outcomes

##### 2.3.1.1. Pain perception and stress relief

39% of music college musicians had perceived pain for 12 months or longer, compared to 32% of university musicians and 22% of university non-musicians. 77.7% of music college musicians and 14.7% of university musicians reported perceiving pain while playing. 2.9% of music college musicians and 40% of university musicians reported very strong perceived pain while playing that stressed them considerably. A post-hoc found that the length of pain perceived (in months) by music college musicians was significantly longer than that perceived by university musicians *t* (58) = 5.87, *p* < .001, *d* = .3 and university non-musicians [*t* (61) = 6.0, *p* < .001, *d* = .4]. 77.1% of music college musicians, 82% of university musicians and 94% of non-musicians followed an active or passive stress relief management plan (e.g., yoga, meditation).

##### 2.3.1.2. Pain catastrophizing

**Figure 1:**
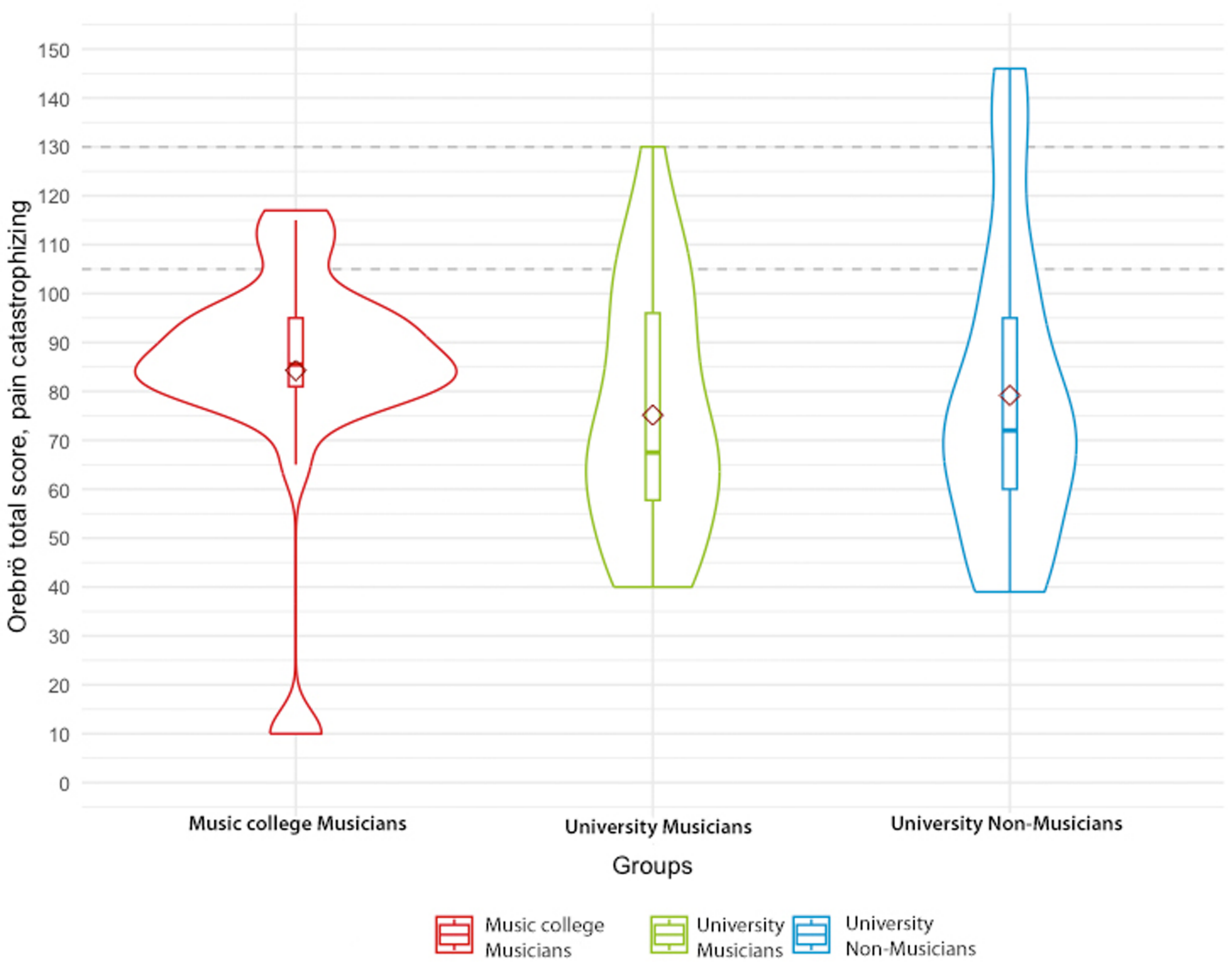
Violin plot for the Orebrö Musculoskeletal pain screening representing the group distribution for music college musicians, university musicians and university non-musicians, including box plot with mean points (diamond shape) and a dotted cut-off lines at ≥105 for moderate and at ≥130 for high pain catastrophising.

Non-musicians had a significantly higher prevalence of high pain catastrophizing (≥130) compared to music college musicians and university non-musicians (*z* = 1.94, *p *= .05). As a reminder, the Örebro notes a maladaptive pain strategy only from a particular score onwards: a score of ≥105 is moderate and ≥130 a high risk of being disabled by pain. Although university musicians showed a lower prevalence of high catastrophizing compared to university non-musicians this was not statistically significant (*p* = .3) (see Figure 1).

##### 2.3.1.3. Depression and anxiety

There was a significant difference in depression prevalence between the music college musicians' group and both the university musicians (*z* = −3.67, *p* = .0002), and the university non-musicians (*z *= 2.16, *p* = .003). Music college musicians showed significantly higher depression prevalence compared to university musicians and non-musicians (see Table 2). The highest anxiety prevalence was found in university non-musicians. When compared to music college musicians, the difference was significant (*z* = −2.01, *p *= .04). There was no statistically significant difference between university non-musicians and university musicians (*p *= .2).

**Table 2:** Results from all standardized tests [mean, standard deviation (SD) and prevalence].

Test	Groups								
	Music College Musicians			University Musicians			University Non-Musicians		
	Mean	SD	Prevalence	Mean	SD	Prevalence	Mean	SD	Prevalence
HADS Anxiety	8.68	3.58	43.7%	4.3	4.05	40.6%	4.5	3.30	55.5%
HADS Depression	7.80	1.4	31.2%	4.41	3.26	9.3%	5.2	3.38	19.4%
MIMS (total score)	49.11	10.42	–	32.5	16.63	–	–	–	–
PSQI	3.37	2.77	10%	–	–	–	–	–	–
CD-9 (total score)	28.6	6.39	93.3%	32.06	16.42	100%	32.9	17.67	90%
Brief Cope active functional	21.65	4.60	–	21.55	4.32	–	20.54	7.11	–
Brief Cope cognitive functional	19.10	5.69	–	17.24	5.60	–	15.96	4.88	–
Brief Cope dysfunctional	9.65	2.64		8.65	2.53	–	8.53	2.72	–
Örebro	82.22	23.32	≥105 = 13.30%	73.97	25.41	≥105 = 13.79%	80.54	30.27	≥105 = 24.13%
			≥130 = 0%			≥130 = 3.4%			≥130 = 10.34%
CBI (Burnout) Personal	2.41	0.29	–	2.89	0.89	–	2.57	0.88	–
CBI Work	3.06	0.88	–	3.03	0.74	–	3.05	1.28	–
CBI Student	3.18	0.37	–	3.56	1.05	–	3.42	1.14	–
CBI Professor	3.88	.01	–	3.76	0.98	–	3.81	0.98	–

Cut-off score for both HADS scales are ≥9; CD-) cut-off is at ≥19.

Except for active functional and cognitive functional scales, higher values indicate more symptoms, less favourable way of dealing with stress (CBI), worse pain catastrophizing and more dysfunctional coping strategies.

For the brief cope active functional and cognitive functional coping scales, higher values indicate a better way of dealing with stress.

##### 2.3.1.4. Depersonalization

There was almost no difference in depersonalization prevalence between college musicians (93.3%), university musicians (100%) and university non-musicians (90%).

##### 2.3.1.5. Coping

An ANOVA found no significant difference between groups for this variable (active functional cope: *p* = .6; cognitive functional cope: *p* = .2; dysfunctional cope: *p *= .7).

##### 2.3.1.6. Burnout

There was no statistically significant difference between groups. The highest level of burnout was experienced based on interactions with teaching staff, followed by fellow students, personal burnout and then work burnout.

##### 2.3.1.7. Sleep

With a poor sleeper prevalence of only 10%, the hypothesis that a lack of sleep would have a negative impact on music college musicians was not supported. However, there was a strong correlation between daytime dysfunction (the need to sleep during the day) and depression [*r_s_* (29) = .91, *p *= .001]. All participants with a bad sleeper score also reported high prevalence in depression, anxiety (<9) and pain catastrophizing (<110).

##### 2.3.1.8. GOLD-MSI and MIMS

**Figure 2:**
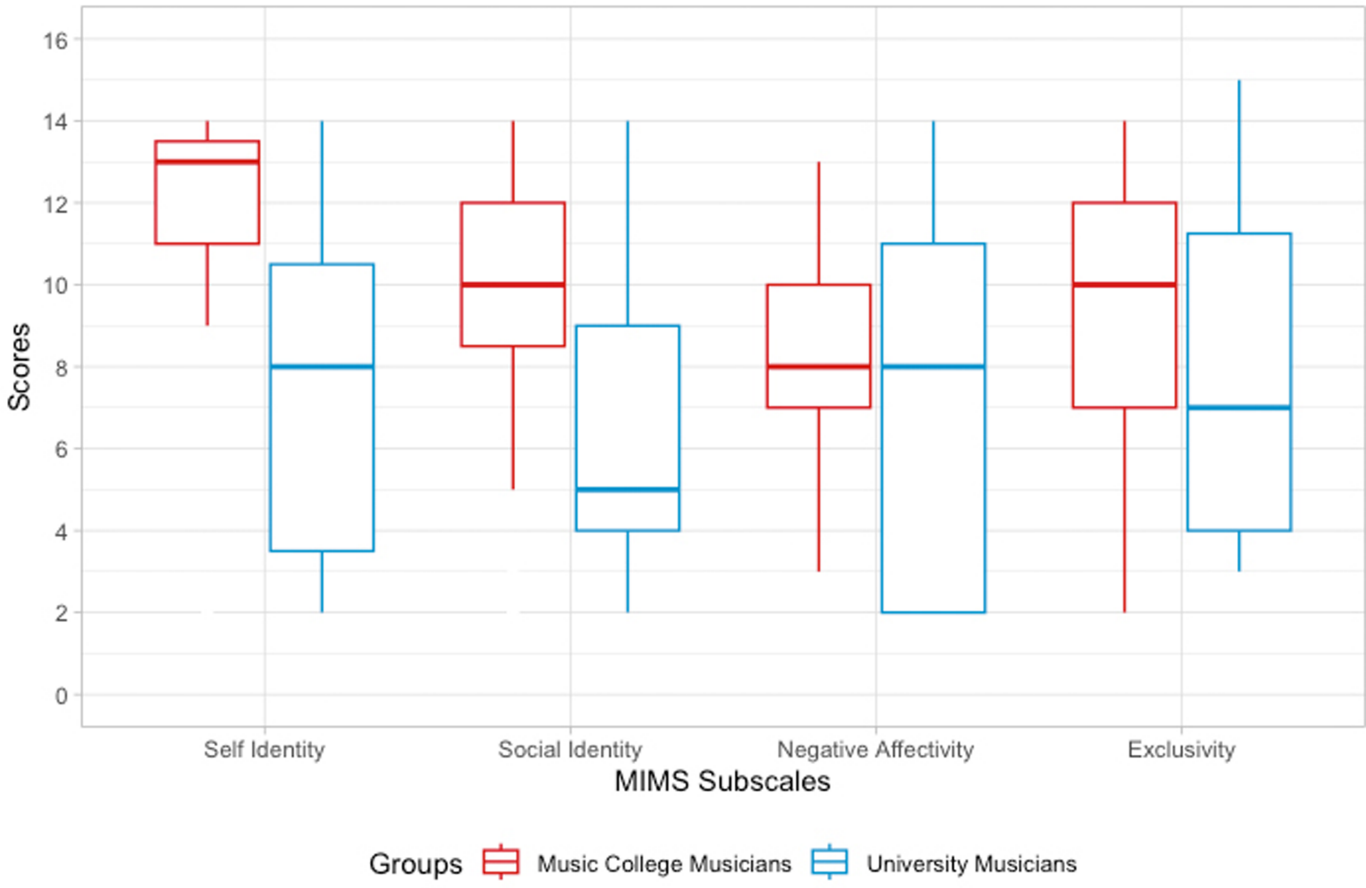
Boxplot for MIMS subscales (self-identity, social identity, negative affectivity, and exclusivity) comparing professional identity for music college musicians and university musicians; significant differences marked by asterisks.

Music college musicians invested more time into daily practice and formal lessons than university musicians but had not accumulated more years of practice. Music college musicians spent more time listening attentively to music and attended more live concerts (audience) than university musicians. Non-musicians took no instrumental or theory lessons. They listened less to music and attended fewer concerts (see Table 2). Moving on to the MIMS, a Mann–Whitney *U*-test determined a significant difference in the full score between music college musicians and university musicians, with a large effect size (*U* = 856.0, *p* = .001, rank-biserial correlation = .57). The subscales self-identity (*U* = 853.0, *p* < .004) and social identity (*U* = 588.0, *p* < .004) were significantly higher. Negative affectivity (*p* = .4) and exclusivity (*p* = .5) did not differ significantly (see Figure 2).

#### 2.3.2. Tree model

**Figure 3:**
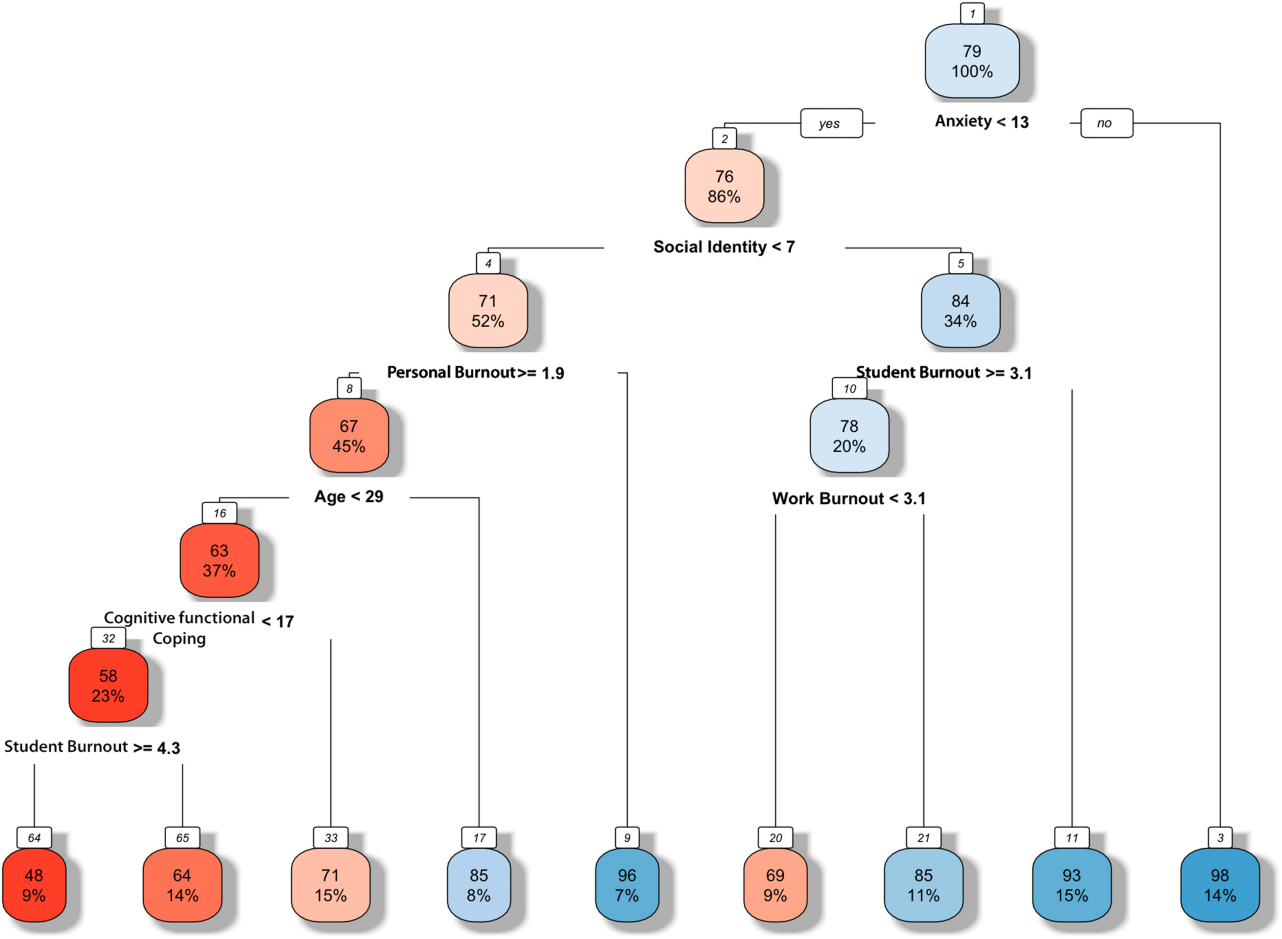
Tree model predicting the combination of variables needed for a high likelihood of pain catastrophizing. Following the boxes from top to bottom, the highest likelihood combination (i.e. the most significant predictor pathway) is anxiety, social identity, personal burnout, < 29 years of age, cognitive functional abilities and burnout with fellow students.

Tree modelling was employed to detect the likeliest combination of variables to predict pain catastrophizing based on all variables (minus sleep) (see Figure 3). The resulting tree model illustrates that pain catastrophizing requires a combination of cognitions/beliefs. Each contributing variable in the tree was further characterized by the degree it was found to have made an impact (e.g., the number from that particular scale). Starting at the top box (anxiety yes/no), our model demonstrates that anxiety on its own does not lead to pain catastrophizing. The pathway to effect requires a further combination of characterized social identity (<7), low personal burnout (>1.9), being younger than 29 years of age, having low cognitive functional abilities (<17) and the experience of a high amount of burnout in relation to fellow students (≥4.3). Low personal burnout referred to a small amount of personal experiences of burnout, such as feeling physically tired or emotionally exhausted. High burnout with fellow students refers to a high amount of feeling burnout stemming from the interaction in university with their fellow students, such as feeling it is hard to work with fellow students.

In summary, non-musicians in our study were significantly more affected by high pain catastrophizing compared to music college musicians and university musicians, despite music college musicians perceiving pain for the longest (12 months and longer) compared to both other groups. More than two thirds of music college musicians reported pain while playing, compared to less than one sixth of university musicians. However, only 2.9% of music college musicians reported very strong pain while playing that they perceived as stressful, compared to 40% of university musicians. Despite pain catastrophizing correlating with depression, anxiety, low cognitive-functional abilities, depression was not a significant factor for pain catastrophizing in the resulting tree model. In terms of music-specific variables, social identity as a musician was found to be a predictive variable however, its presence required a combination of various other variables to become a valid predictor.

## 4. Discussion

To gain a better understanding of the chronic pain experience in student musicians, this study investigated pain catastrophizing alongside seven complementary variables: anxiety, depression, depersonalisation, burnout, coping strategies, professional identity, and sleep. The results of the student musicians were compared to those reported from student non-musicians to identify any unique pathways to effect predicted by group membership.

Our tree model confirmed the biopsychosocial model from the chronic pain literature, which requires a combination of variables to cause a maladaptive process: anxiety, social identity, personal burnout, age, low cognitive functional abilities, and burnout with fellow students. Group testing, however, showed that despite having perceived pain for significantly longer, music college musicians reported the lowest pain catastrophizing prevalence. In other words, pain catastrophizing was significantly lower in music college musicians compared to their university non-musician peers. Furthermore, music college musicians showed a significantly lower anxiety prevalence compared to university non-musicians. Finally, they reported higher satisfaction in their studies (job satisfaction) and fewer missed (work) days due to pain-based issues, when compared to their non-musician peers.

On the surface, these findings appear to disagree with much of the specialist literature on musicians' health, which claims that musicians' pain significantly decreases their wellbeing (38). However, chronic pain, as previously stated, is not simply acute pain plus time, but a separate condition. In chronic pain, pain behaviors and cognitions lead to substantial cortical plasticity alterations that could be viewed as “pain memories”, influencing painful and non-painful processing in the somato-sensory system. While pain in adolescence is generally under-researched, even more so in young musicians, Zamorano et al. (18), have demonstrated distinct pain-related cortical plasticity changes in musicians. When considering the duration of the perceived pain in isolation, one could suppose that musicians would use maladaptive strategies that lead to such neurological changes. However, the long career of an average classical musician, combined with the high job satisfaction, especially in older musicians, call this hypothesis into question (16).

Before we consider the role of professional identity as one possible variable, we need to come back to basic pain management prognostics. There is reliable evidence that children with maladaptive coping strategies are more likely to perceive more pain when they reach adolescence. This trend increases even more from adolescence to adulthood (39). In other words, as a group, it is highly unlikely that student musicians with maladaptive pain coping strategies might end up with a long career in music and high job satisfaction. While pain-induced beliefs and cognitions and neurological changes can be altered and even reversed, it is statistically unlikely to happen to an entire group. Furthermore, chronic pain processes are neither fast nor are they conscious decisions, but complex processes that grow and are reinforced over the course of a lifetime, thus have a tendency to increase with age. However, while age can be an indicator for pain catastrophizing, it should not be viewed in isolation, but rather in combination with other variables such as anxiety, (self) efficacy and/or other coping abilities, to carry reasonable significance.

In line with the relevant literature on chronic pain, our tree model underscored that social identity constitutes an important variable in the process of pain catastrophizing (40). Music college musicians showed significantly higher scores in social and self-identity than university musicians. Additionally, they differed from the latter group by allocating more time to their daily practice. Both groups invested an equal number of years into their practical and theoretical musical training. Most instruments require years of practice to be mastered at an elite level required for instance by a music college. We can therefore infer that both groups followed a similar education until reaching college or university level. Moreover, we can assume that during these formative training years young musicians got accustomed to a degree of performance-related pain. Professional identity not only reflects a person's personal attitudes but is also fed by the environment (41) Thus, their pain-enduring beliefs began early in musical training, and were, most probably, reinforced by parents, study friends, teachers and/or coaches.

As a side note, the hypothesis that musicians' experience of pain depends in part on their personality (42) should be viewed in a larger context of chronic pain. Here research points out that tying chronic pain to personality-based models should be most critically evaluated as there is little empirical evidence (43). Moreover, such an approach could lead to further stigmatization of individuals. In this context, personality-based models have a historical importance but going forward, more contemporary approaches using interpersonal models are more appropriate.

One conclusion we can draw is that perceived pain did not have an impact on students' education goals. Life goals, such as becoming a professional (elite) musician, were still pursued. Furthermore, we can draw the conclusion that the degree of higher education and/or place of study make a difference for pain catastrophizing in student musicians. We know that both music college musicians and university musicians invested years into the practice of their instruments/voice. For reasons of their own, they decided to either apply for music college, to follow a more academic path at a university, or to completely change their main study subject. Their professional identity modified in line with these career choices, similarly to the professional identity of college athletes whose professional identity was found to decline after they chose a different career path to sports (44, 45). Further research is needed to understand the developmental aspects of this identity process more fully.

Two other important variables that are discussed in regards to musicians and pain are depression and anxiety (45). The chronic pain literature describes both variables as having distinct properties that can lead to maladaptive pain perception processes. High anxiety is a reliable variable carrying considerable weight for maladaptive pain processes (46). Our study supports these findings, the university non-musicians showed a significantly higher anxiety prevalence compared to the two other groups, and consequently had the highest prevalence in pain catastrophizing.

Depression did not carry such a significance. Music college musicians had significantly higher depression prevalence compared to university non-musicians yet showed significantly lower levels of pain catastrophizing. While more studies are needed to fully understand this variable, we can extrapolate from studies into chronic back pain and depression. These found that depression only led to pain catastrophizing if the individuals had a mild depression on the day of the surgery (43). This also brings back the importance of the factors time and degree of a specific variable (42, 43).

It is important to note the limitations of this study to allow a meaningful interpretation. Firstly, despite providing more than the number of participants required for meaningful results, the overall sample size is modest, which reflects the exploratory nature of the study. This could be addressed in future studies with larger numbers of participants. Secondly, and in keeping with the literature, some participants declined to disclose information about their mental health status (47). Thirdly, for anonymity reasons, variables such as gender were excluded. Specifically, pilot work indicated that music students in particular feared to be identified when reporting pain or mental health issues, and hence any variables without strong predictive value were excluded in order to offer increased safeguarding. Fourthly, this study was designed based on empirical findings from studies into musicians' pain perception. We did not anticipate that our findings would contrast with most of the current literature. In hindsight, it would have further elucidated our findings, had we included additional instruments for pain processing to help interpret the data. Depending on the scope this could include questionnaires on the (predisposition of) performance anxiety, super-sensitivity or other underlying medical conditions. (e.g., migraine). We would suggest that future studies take this into account as they explore this area in more detail (e.g., development and degree of chronification or family history).

Overall, our findings provide several insights for chronic pain research in student musicians. Despite long-term perceived chronic pain (12 months and longer), music college musicians reported the best pain management strategies out of the three groups. Maladaptive strategies for chronic pain presented a larger problem for university non-musicians. This group showed the highest prevalence, increasing the likelihood of an overall negative long-term impact on their work, life, and wellbeing. The difference we found between music college musicians and university musicians in pain catastrophizing levels point to the conclusion that environment shapes how chronic pain is managed. General pain chronification predictors all played a role, independent of whether the participant is a student musician or not with the exception of the depression variable, which requires further investigation into its role in chronic pain.

We conclude on the critical point that, whilst we found that music college musicians generally have good chronic pain management strategies, this should not minimize the fact that, as a group, they are disproportionately affected by chronic pain. Chronic pain management guidelines stipulate that, in contrast to the treatment of acute pain, a pain-free status may not be obtained (48). Moreover, pain control needs to be optimized and adverse outcomes minimized, such that quality of life may be enhanced. The implication for musicians is that pain prevention methods should be introduced at the earliest stages of training. Hopefully in this regard, the chronic pain literature suggests that low scores in pain catastrophizing make individuals more open to pain preventative methods, as well as making compliance more likely (43). Hence a proactive and informed approach to early pain management during musical training, informed by the multiple predictive factors identified in this article, holds the promise for a future where at-risk musicians are identified sooner and offered the strategies that will enhance their long-term career prospects and wellbeing.

## Data Availability

The raw data supporting the conclusions of this article will be made available by the authors, without undue reservation.
